# Evaluation of Posaconazole Pharmacokinetics in Adult Patients with Invasive Fungal Infection

**DOI:** 10.3390/biomedicines5040066

**Published:** 2017-11-20

**Authors:** Sarah Allegra, Giovanna Fatiguso, Silvia De Francia, Fabio Favata, Elisa Pirro, Chiara Carcieri, Amedeo De Nicolò, Jessica Cusato, Giovanni Di Perri, Antonio D’Avolio

**Affiliations:** 1 Department of Medical Sciences, Unit of Infectious Diseases, University of Torino, ASL Città di Torino, Amedeo di Savoia Hospital, Corso Svizzera 164, 10149 Turin, Italy; giofatiguso@gmail.com (G.F.); fabio_favata@libero.it (F.F.); chiaracarcieri@gmail.com (C.C.); amedeo.denicolo@unito.it (A.D.N.); jessica.cusato@unito.it (J.C.); giovanni.diperri@unito.it (G.D.P.); antonio.davolio@unito.it (A.D.); 2 Department of Biological and Clinical Sciences, University of Turin, S. Luigi Gonzaga Hospital, 10043 Orbassano (TO), Italy; silvia.defrancia@unito.it (S.D.F.); elisa.pirro@unito.it (E.P.)

**Keywords:** therapeutic drug monitoring (TDM), triazoles, HPLC, antifungal, invasive fungal infections (IFIs)

## Abstract

Mortality and morbidity due to invasive fungal infections have increased over the years. Posaconazole is a second-generation triazole agent with an extended spectrum of activity, which shows a high interindividual variability in its plasma levels, rendering dosing in many patients inconsistent or inadequate. Hence, posaconazole therapeutic drug monitoring, which is easily available in clinical practice, may improve treatment success and safety. The aim of the study was to describe posaconazole pharmacokinetics, and to evaluate the utility of therapeutic drug monitoring for therapy and prophylaxis in a cohort of adult patients. A fully validated chromatographic method was used to quantify posaconazole concentration in plasma collected from adult patients at the end of the dosing interval. Associations between variables were tested using the Pearson test. The Mann-Whitney test was used to probe the influence of categorical variables on continuous ones. A high inter-individual variability was shown. Of the 172 enrolled patients, among those receiving the drug by the oral route (*N* = 170), gender significantly influenced drug exposure: males showed greater posaconazole concentration than females (*p* = 0.028). This study highlights the importance of therapeutic drug monitoring in those with invasive fungal infections and its significant clinical implications; moreover we propose, for the first time, the possible influence of gender on posaconazole exposure.

## 1. Introduction

Mortality and morbidity due to invasive fungal infections (IFIs) has increased over the years, despite the development of better and faster diagnostic methods and the availability of antifungal treatments [1]. IFIs are a major cause of life-threatening diseases in immunocompromised patients, including cancer patients receiving chemotherapy, hematopoietic stem cell and solid organ transplant recipients, HIV positive patients, those receiving invasive clinical procedures or patients hospitalized in intensive care units [2,3]. Moreover, emerging opportunistic fungal pathogens are now significantly prevalent in patients receiving massive antifungal treatment [4]. The introduction of the echinocandins and triazoles improved the therapeutic options. There are five classes of antifungal agents currently in use for treatment of IFIs: polyenes (amphotericin, nystatin), allylamines (terbinafine), azoles (fluconazole, itraconazole, voriconazole, posaconazole, isavuconazole), pyrimidine analogous (5-fluorocytosine), and echinocandins (micafungin, caspofungin, and anidulafungin) [5,6]. Posaconazole (PSC; Noxafil^®^) is a second-generation triazole agent with an extended spectrum of activity. It is used for the treatment of IFIs and is recommended as a first-line prophylaxis during prolonged neutropenia, leukaemia induction treatment and graft-versus-host disease [7]. Particularly, PSC is used to treat infections including *Candida* spp., *Aspergillus* spp., *Cryptococcus neoformans* and the Mucorales; moreover, it is used in fusariosis as salvage therapy, in invasive aspergillosis patients resistant or intolerant to first-line agents, for chromoblastomycosis and mycetoma resistant and/or intolerant to itraconazole, and for coccidioidomycosis resistant and/or intolerant to amphotericin, itraconazole or fluconazole [8]. PSC shows a linear pharmacokinetics with daily doses up to 800 mg; further dose increases do not result in proportional increases in drug exposure [9]. It has poor water solubility, thus necessitating ingestion with a high-fat meal, and is absorbed at low intestinal pH [10]. PSC shows a time of 5 h to reach the maximum serum concentration of and a half-life of about 34 h (1 week) [11]. The drug is metabolized primarily by glucuronidation, rather than oxidation; it is a cytochrome (CYP) 3A4 activity inhibitor [12]. PSC is available as a solid tablet, oral suspension (OS) and intravenous (IV) formulation. The oral bioavailability of tablets and capsules is better than the suspension, although considerable variability is still seen, suggesting that therapeutic drug monitoring (TDM) should be considered [13]. The tablet and oral suspension formulations of PSC are not considered interchangeable, due to different dosing and pharmacokinetics [14]. For patients with established disease, the probability of a clinical response increases with increasing drug exposure [15]. TDM may be an important tool for maximizing efficacy [16]. Drug levels and treatment outcomes depend on host factors, target organisms and associated interventions, and TDM can guide timely and appropriate drug dosage modifications [17]. Published clinical TDM studies have been conducted, and have observed that PSC dose modifications can result in more appropriate drug plasma levels [18,19,20]. The guidelines recommend PSC concentrations ≥1000 ng/mL during treatment and ≥700 ng/mL for prophylactic use [21,22]. Lower concentrations have been associated with breakthrough IFIs; although an upper boundary of 3500 ng/mL is suggested for the average PSC levels [23].

The aim of this study was to describe PSC pharmacokinetics and to evaluate the utility of PSC TDM for therapy and prophylaxis in a cohort of adult patients.

## 2. Results

One hundred and seventy-two adult patients (96 males, 55.8%) treated with PSC were enrolled. The majority (93.0%; *N* = 160) were caucasian. Sixty-eight (39.5%) received PSC antifungal prophylaxis. Routes of administration were OS (*N* = 170; 98.8%) or IV (*N* = 2; 1.2%). Mean, SD, median and interquartile range 25th to 75th percentiles (IQR) values for age, BMI and PSC plasma concentration are compared in Table 1.

The drug dosage was evaluated with a score from 1 to 12: 100 mg twice daily (t.d.) (1), 100 mg three times daily (th.d.) (2), 200 mg once daily (o.d.) (3), 200 mg t.d. (4), 200 mg th.d. (5), 300 mg t.d. (6), 300 mg th.d. (7), 400 mg o.d. (8), 400 mg t.d. (9), 400 mg th.d. (10), 500 mg t.d. (11) and 800 mg o.d. (12) (Table 2).

**Figure 1:**
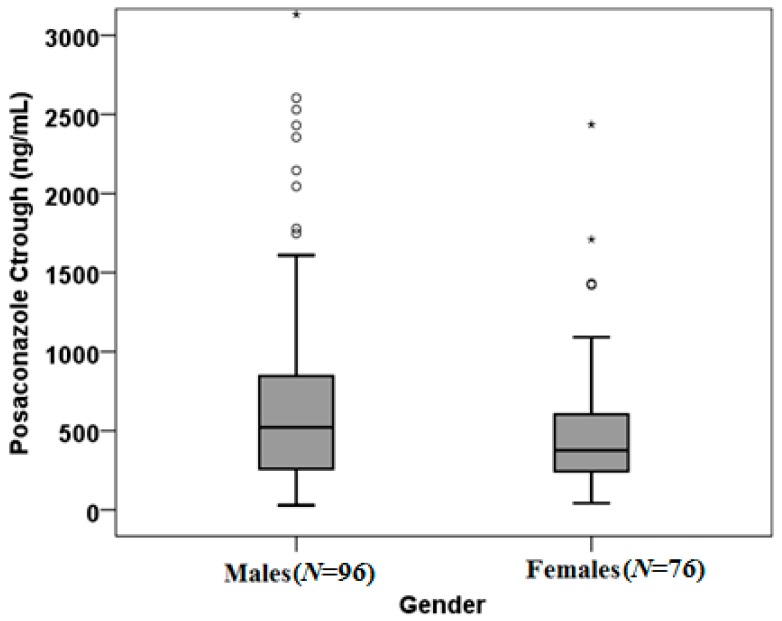
Plots of gender influence on posaconazole trough concentration, considering all the 172 enrolled patients (*p* = 0.028). Boxes and black lines in boxes represent respectively interquartile ranges (IQR) and median values; open dots and stars represent outlier values. Median values (horizontal line), interquartile range (IQR, bars), patient values (black square), highest and lowest value (whiskers) are shown. Males (*N* = 96) had 521.50 ng/mL (IQR: 256.00–240.25 ng/mL) median concentrations; Females (*N* = 76) had 376.50 ng/mL (IQR: 240.25–376.50 ng/mL) median concentrations.

Evaluating those receiving PSC treatment based on published PSC through levels cut-offs [21,22,23], we observed that 84 patients (80.8%) showed sub-optimal exposure (drug concentrations <1000 ng/mL), 18 (17.3%) had concentrations higher than the efficacy cut-off (drug concentrations ≥1000 ng/mL) and 2 (1.9%) had toxic drug levels (drug concentrations ≥3500 ng/mL). Instead, for prophylaxis, 50 patients (61.7%) showed trough levels lower than the efficacy cut-off (drug concentrations <700 ng/mL), 30 (37%) had concentrations included in the efficacy range (drug concentrations ≥700 ng/mL) and 1 (1.2%) had drug levels higher than the toxicity cut-off levels (drug concentrations ≥3500 ng/mL). A high interindividual variability was found between PSC Ctrough: the median value was 419.50 ng/mL and the IQR range was 252.50 and 778.75. Mann-Whitney U test showed a significant influence of gender on drug exposure (*p* = 0.028): males (*N* = 96) had 521.50 ng/mL (IQR: 256.00–240.25 ng/mL) median concentrations, while females (*N* = 76) had 376.50 ng/mL (IQR: 240.25–376.50 ng/mL) (Figure 1).

## 3. Discussion

IFIs are still a leading cause of morbidity and mortality; they occur in a setting of multiple morbidities, and are associated with fatality rates of 30–70% [1]. Azoles remain the corner-stone of prevention and treatment of IFIs, including acute invasive aspergillosis [24]. However, the clinical use of these drugs is characterized by frequent pharmacological drawbacks in terms of pharmacokinetic variability and drug–drug interactions [25]. PSC is a third-generation triazole antifungal agent, structurally similar to itraconazole, with a broad spectrum of activity; it is registered for use in humans, and is available as a 40-mg/mL oral suspension, as delayed-release tablets, and as an IV infusion [26]. TDM, a dosage individualization strategy, could help to minimize toxicity whilst maximizing the efficacy of PSC [11]. In this study, we analyzed the pharmacokinetics of PSC in patients with IFIs receiving PSC therapy. Our results show that PSC exposure has a high interindividual variability. Participants’ age, BMI, and PSC administered dose did not significantly affect PSC pharmacokinetics. On the contrary, an inverse relationship among age and PSC volume of distribution was shown in a study regarding prophylactic PSC use in patients undergoing allogeneic hematopoietic stem cell transplantation [27], and a relationship between weight and a larger PSC volume of distribution was observed in a PSC population pharmacokinetic analysis [28]. Considering the gender effect on drug exposure, we observed that males had higher median PSC Ctrough values than women (*p* = 0.028; Figure 1). Gender-related differences, such as body size and muscle mass, may result in drug pharmacokinetic differences, as reported by Beierle et al. [29]. Although various studies have not observed an influence of gender on PSC pharmacokinetics [30], this factor could alter the disposition of other triazole antifungal agents: voriconazole plasma levels were twofold higher for healthy women than for healthy men in the same age range [31]. Moreover, sex-based differences in drug metabolism could be due to differences in the hepatic enzymes expression, including CYP (such as the CYP3A4). Indeed, the sex-related dimorphic expression of *CYPs*, and other genes expressed in liver, depends on the growth hormone plasma levels emitted by the pituitary gland, which controls sexual maturation. Our study has some limitations. It has a retrospective design, it lacks a standardized protocol for PSC dosing, and we included a limited patient sample size; therefore, further research applied to larger cohorts is required to confirm the reported data. This study highlights the importance of TDM in patients with IFIs, and its significant clinical implications; moreover, we propose, for the first time, the possible influence of gender on PSC exposure. The results from the present study might be further explained through pharmacogenetic analyses [32].

## 4. Material and Methods

### 4.1. Patients and Inclusion Criteria

Plasma samples were collected at the Laboratory of Clinical Pharmacology and Pharmacogenetics (Department of Medical Sciences, Unit of Infectious Diseases, University of Turin, Amedeo di Savoia Hospital, Turin) and Clinical Pharmacology Service “Franco Ghezzo”(Department of Biological and Clinical Sciences, University of Turin, S. Luigi Gonzaga Hospital) from different Hospitals in Piedmont (Italy). Inclusion criteria were: age above 18 years old, diagnosed IFI, treatment with PSC for prophylaxis or therapy purposes, and an adherence of 90%. Patients on treatment with potential interacting drugs, allergy or intolerance to PSC, HIV infection, severe malnutrition, liver cirrhosis, chronic renal failure (with estimated creatinine clearance, eCRCl < 60 mL/min) were excluded. Study protocol (“PkPG_J02AC Studio retrospettivo per la valutazione e farmacocinetica e farmaco-genetica della terapia antimicotica con farmaci triazolici”) was approved by the local Ethics Committee in accordance with the Declaration of Helsinki. Written informed consent for the study was obtained from each enrolled subject. For all patients, the following data were available: gender, age, body mass index (BMI), ethnicity and PSC dose.

### 4.2. Determinations of Posaconazole Plasma Concentration

Blood samples were taken immediately before drug intake (Ctrough), under steady-state conditions. Plasma samples were obtained by centrifugation at 3000 rpm for 10 min at 4 °C. 6,7-dimethyl-2,3-di(2-pyridyl) quinoxaline (QX), used as the internal standard (IS), was purchased from Sigma-Aldrich Corporation (Milan, Italy), and PSC was purchased from Sigma-Aldrich Corporation (Milan, Italy). Acetonitrile (HPLC grade) and methanol (HPLC grade) were purchased from VWR (Milan, Italy). Formic acid was from Sigma-Aldrich Corporation (Milan, Italy). HPLC-grade water was produced by a Milli-DI system coupled with a Synergy 185 system by Millipore (Milan, Italy). Plasma samples (200 μL) have been pipette in a polytetrafluoroethylene tube with 50 μL of IS. A simple protein precipitation (using 200 μL) was used to extract drug from samples. Each sample was vortexed for 15 s and centrifuged at 12,000 rpm for 10 min (4 °C). One hundred μL of supernatant, diluted with 100 μL of water, was transferred to a glass vial. A part of the sample (50 μL) was injected into the HPLC-MS system. All extraction procedures were carried out at room temperature. The HPLC-MS system used was a Waters system (Milford, MA, USA) with a binary pump (1525), in-line degasser AF, 717-plus autosampler, and Micromass ZQ mass detector. The software used was LC-MS Empower 2 Pro (version year 2005; Waters) [33,34]. The chromatographic separation was carried out at 35 °C using a column oven on a C18 Atlantis T-3 5-μm column (150 mm by 4.6 mm, inside diameter (i.d.)) (Waters, Milford, MA, USA), protected by a Security Guard with a C18 precolumn (4.0 mm by 3.0 mm, i.d.) (Phenomenex; Torrance, CA, USA). The mobile phase (50:50 water with formic acid (0.05%)/acetonitrile with formic acid (0.05%)) was ramped to 20:80 within 6.5 min. The flow rate was set at 1 mL/min. Detector settings were: electrospray ionization (ESI+), capillary voltage (3.5 kV), source temperature (110 °C), desolvation temperature (350 °C), nitrogen desolvation flow (400 L/h) and, nitrogen cone flow (50 L/h). The ion m/z values monitored were: 351.0 for PSC and 313.4 for QX, cone voltage was 25 V and 50, respectively. This work was carried out in a PHASE I AIFA, UNI EN ISO 9001:2008 and 13485:2012 (CE-IVD) certified laboratory.

### 4.3. Statistical Analysis

For descriptive statistics, continuous and non-normal variables were summarized as average, standard deviation (SD), median and interquartile range (IQR); 25th to 75th percentiles were calculated to measure the statistical dispersion of the data; categorical variables were summarized as frequency and percentage. The Shapiro-Wilk test was used to evaluate normality for all variables. The Kolmogorov-Smirnov test was performed to define the correspondence of each parameter with a normal or non-normal distribution. The Independent Samples *t* Test was used to compare the means of two independent groups, considering the level of statistical significance (*p* value < 0.05). The Pearson linear correlation coefficient (r) was used to investigate the strength of the association between two quantitative variables considering the level of statistical significance (*p* value < 0.05). The Mann-Whitney *U* test was used to probe the influence of categorical variables on continuous ones, considering the level of statistical significance (*p* value < 0.05). All tests were performed with IBM SPSS Statistics 22.0 for Windows (Chicago, IL, USA).

## Figures and Tables

**Table 1:** Mean, standard deviation, median and interquartile range for age, body mass index and posaconazole plasma concentration.

Variable	*N* = 172			
	Mean	Standard Deviation	Median	IQR
Age (years)	47.14	18.952	49.50	27.00–64.00
BMI Kg/m^2^	24.49	4.342	24.16	21.83–27.01
PSC C_trough_ ng/mL	726.71	914.443	419.50	252.50–778.75

**Table 2:** Number and percentage of patients for each dose regimens.

*N* = 172			
PSC Dose	Dose Score	*N*	%
100 t.d.	1	1	0.6
100 th.d.	2	1	0.6
200 o.d.	3	27	15.7
200 t.d.	4	84	48.8
200 th.d	5	13	7.6
300 t.d.	6	1	0.6
300 th.d.	7	2	1.2
400 o.d.	8	1	0.6
400 t.d.	9	30	17.4
400 th.d.	10	7	4.1
500 th.d.	11	3	1.7
800 o.d.	12	2	1.2
